# A novel biweekly pancreatic cancer treatment schedule with gemcitabine, 5-fluorouracil and folinic acid

**DOI:** 10.1038/sj.bjc.6601045

**Published:** 2003-07-15

**Authors:** P Correale, S Messinese, S Marsili, F Ceciarini, D Pozzessere, R Petrioli, M Sabatino, D Cerretani, M Pellegrini, T Di Palma, A Neri, A Calvanese, E Pinto, G Giorgi, G Francini

**Affiliations:** 1 Oncology Section, Department of Human Pathology and Oncology, Siena University School of Medicine, Viale Bracci 11, 53100 Siena, Italy; 2 ‘Giorgio Segre’ Pharmacology Department, Italy; 3 Division of Surgical Science, Faculty of Medicine, University of Siena, Italy; 4 Oncology Operative Unit, Casa di Cura “Tortorella”, Salerno, Italy

**Keywords:** Pancreatic adenocarcinoma, gemcitabine, folinic acid, 5-fluorouracil, biweekly schedule

## Abstract

Pancreatic adenocarcinoma is a common disease considered to be poorly responsive to antiblastic treatment. Recent clinical and preclinical results suggest that a combined treatment of gemcitabine (GEM), 5-flurouracil (5-FU) and folinic acid (FA) offers a clinical benefit in patients with advanced pancreas adenocarcinoma. The aim of this phase II clinical trial was to evaluate the antitumour activity and toxicity of a novel biweekly schedule of this combination in patients with pancreatic adenocarcinoma. A total of 42 patients received a 30 min infusion of FA (100 mg m^−2^) and 5-FU (400 mg m^−2^) (FUFA) on days 1–3, and GEM 1000 mg m^−2^ on day 1 every 15 days. We observed 13 objective responses (two complete, 11 partial) and 23 stable diseases. The median time to progression was 9.75 months (95% Confidence Interval (CI), 6.88–12.62) and the median overall survival was 13.10 months (95% CI 9.64–16.56). There were seven cases of each grade III gastroenteric and haematological toxicity. The GEM plus FUFA combination appears to be well tolerated and very active in patients with pancreatic carcinoma.

Pancreatic adenocarcinoma is one of the most detrimental gastroenteric malignancies and the fourth–fifth leading cause of death from malignancies in Western countries (Silverberg *et al*, 1990; Bramhall *et al*, 1995; Rocha Lima and Centeno, 2002). Radical surgery in the low-stage disease is considered the only chance of cure, but applies to less than 5% of the patients; unfortunately, the majority of them are in an advanced stage at the time of the diagnosis and only palliative therapies can be adopted with minimal or no impact on the survival which remains very poor (Bakkevold *et al*, 1992; Bramhall *et al*, 1995; Rosewicz and Wiedenmann, 1997; Rocha Lima and Centeno, 2002).

Chemotherapy has long been considered largely ineffective, but a cautious optimism has recently been expressed since the discovery of new drugs such as gemcitabine (GEM), a fluorinated nucleoside, which used alone or in combination with 5-fluorouracil (5-FU), has been considered effective in terms of antitumour activity and palliation in these patients (Cohen *et al*, 2002; Heinemann, 2002; Jacobs, 2002; Oettle and Riess, 2002; Evans *et al*, 2001). Both 5-FU and GEM are fluoropyrimidine prodrugs that need to be converted to cytotoxic metabolites in the tumour cells in order to exert their antitumour activity; several authors have proposed that the two drugs may interact along their respective pathways of activation leading to a synergistic antitumour activity against a number of different malignancies (including pancreatic carcinoma) *in vitro* and *in vivo* (Plunkett *et al*, 1996; Allegra and Grem, 1997; Hidalgo *et al*, 1997,Hidalgo *et al*, 1999; Berlin *et al*, 1998–99; Correale *et al*, 1999; Schulz *et al*, 1998; Chu *et al*, 2001), and for this reason the combination of the two drugs has been investigated in a number of clinical trials in patients with advanced pancreatic carcinoma (Grem, 1996; Hidalgo *et al*, 1997,Hidalgo *et al*, 1999; Berlin *et al*, 1998–99,Berlin *et al*, 2002; Cascinu *et al*, 1999; Correale *et al*, 2000; Oettle and Riess, 2002). In previous studies of our groups, we demonstrated that GEM affects 5-FU pharmacodynamics (Correale *et al*, 1999) and pharmacokinetics, (Correale *et al*, 2003) and determines synergic antitumour activity *in vitro* (Correale *et al*, 1999). The results of a previous phase I–II clinical trial involving patients with different malignancies (including pancreas and colorectal cancer) suggested that the combination is active as a second-line chemotherapy in advanced pancreatic carcinoma (Correale *et al*, 2000).

On these bases, we have designed the present phase II clinical trial in order to investigate the antitumour activity and toxicity of a novel biweekly schedule of treatment combining GEM, with 5-FU and FA (FUFA) in patients with advanced pancreatic carcinoma.

## MATERIALS AND METHODS

*Eligibility criteria*: The inclusion criteria required a histological diagnosis of pancreatic adenocarcinoma, an Eastern Cooperative Oncology Group (ECOG) ⩽2, a life expectancy of >3 months, normal renal and hepatic function, white blood cell count >2500 mm^−3^, haemoglobin >9 g dl^−1^, platelet cell count >100 000 mm^−3^, and normal cardiac function. The exclusion criteria were any major organ failure, central nervous system involvement, second tumours, or active infectious disease. The study was approved by a local (University) ethics committee. All patients gave their written informed consent. The study was designed to test the hypothesis that the combination of GEM plus FUFA is active in the treatment of pancreatic carcinoma. A minimum of 25 patients was required to maintain an alpha and beta error of, respectively, 0.05 and 0.2. If no clinical response could be demonstrated in the first 10 consecutive patients, the study was to be terminated early.

Patients received FA (100 mg m^−2^) followed by 5-FU (400mg/m^−2^) intravenous infusion (FUFA) on days 1–3. GEM (1000 mg m^−2^) was administered on day 1 (before FA and 5-FU infusion). The cycles were repeated every 15 days. These doses were extrapolated from previous studies of FUFA alone or in combination with GEM.

*Clinical assessments*: A complete history, physical examination, complete blood count, and serum chemistry were performed before the start of the treatment and repeated every month. Complete disease staging was undertaken at baseline, and after 6- and 12-treatment cycles by chest X-ray, computed tomography, and ultrasound scans. Response and toxicity were assessed according to standard World Health Organization (WHO) criteria (World Health Organization, 1979).

*Statistical analysis*: Overall survival and time to progression were calculated by performing the Kaplan-Meier curves.

## RESULTS

### Patient characteristics

A total of 42 patients with histologically confirmed, unresectable pancreatic adenocarcinoma (28 males and 14 females; average age of 61 years: range 31–81) were included in the study between October 1999 and April 2002. In all, 15 patients underwent palliative surgery for obstructive jaundice or duodenal obstruction before beginning the chemotherapy. In all, 11 patients had locoregional unresectable disease while 31 had metastatic disease (22 with liver metastases and nine with metastases not involving the liver).

None of the patients had received previous chemotherapy, radiotherapy or chemoradiotherapy. Table 1

**Table 1:** 

**Characteristics**	**Number of patients**
Patients evaluable for response	42
Patients evaluable for toxicity	42
Age (years)	
Median	61
Range	32–81
Sex	
Male	28
Female	14
Performance status (ECOG)	0–2
Histology	
Pancreatic adenocarcinoma (G2-3)	42
Stage	
III	11 (26.2%)
IV	31 (73.8%)
Previous treatment	
Surgery	15 (35.7%)
None^a^	27 (64.3%)
Disease extension	
Locoregional	11 (26.2%)
Metastatic (A+B)	31 (73.8%)
A Liver	22 (52.4%)
B No liver (bone+lung+peritoneum)	9 (1+2+6) (21.4%)

a Histological diagnosis performed by ultrasound-guided thin-needle biopsy.

summarises their main characteristics.

A total of 336 cycles of treatment were administered (median: range of 8 cycles per patient: range 2–12).

### Toxicity profile

The treatment seemed to be very well tolerated; no WHO grade IV toxicity or toxic death was recorded. The most frequent side effects involved gastroenteric (seven cases, grade II–III) or heamatological toxicity (seven cases, grade III), none of which required treatment discontinuation or delays. Moderate asthenia and fatigue were reported by 10 patients during the second week of treatment.

None of the patients experienced anaemia, increased creatinine/blood urea nitrogen levels, or hypotension.

### Response and survival

All patients were enrolled with intent to treat and were evaluable for response. In this study, we recorded an objective response in 13 out of 42 patients (two complete (4.8%) and 11 partial responses (26.2%)); and a disease stabilisation in 23 (55%) cases; one patient was prematurely withdrawn from the study because of the rapid deterioration in *performance status* due to very rapid progression of disease, while five of them experienced a rapid disease progression within the first six cycles. The time to progression of these patients was 9.75 months (95% of confidence internal (CI) 6.88–12.62).

The treatment also appeared to have a considerable impact on patient survival: the overall survival was in fact 13.1 months (95% CI 9.64–16.56) with four patients alive after 42 months after the diagnosis.

Two patients, who developed a single metastatic lesion in the liver 6 months after the end of the chemotherapy underwent partial hepatectomy and a further six chemotherapy cycles using the same schedule. They are still disease free, respectively, 6 and 8 months after the metastatic resection.

## DISCUSSION

The vast majority of pancreatic carcinoma patients are diagnosed with locally advanced or metastatic disease that precludes surgical resection and needs systemic treatment (Rosewicz and Wiedenmann, 1997). Before GEM became available for clinical use, a large number of trials of other drugs or drug combinations had reported a high level of toxicity with minimal antitumour activity and no significant gain in median survival, which ranged between 5 and 6 months even in the most optimistic studies (Mallinson *et al*, 1980; Friedman *et al*, 1981; Cullinan *et al*, 1985; Palmer *et al*, 1994; Lionetto *et al*, 1995; Evans *et al*, 2001). GEM was the first cytotoxic drug to be approved in the US as a first-line treatment for pancreatic carcinoma. It was effective in controlling cancer-related symptoms, but still led to a low rate of objective responses, and did not affect the median patient survival in comparison with 5-FU alone (Burris *et al*, 1997). Further studies testing different schedule of GEM administration (Ulrich-Pur *et al*, 2000) or combination of GEM with other drugs such as 5-FU (Schulz *et al*, 1998; Berlin *et al*, 1998–99,Berlin *et al*, 2002; Cascinu *et al*, 1999; Hidalgo *et al*, 1999; Cohen *et al*, 2002; Heinemann *et al*, 2000; Oettle and Riess, 2002), cisplatinum (Colucci *et al*, 1999, Heinemann *et al*, 2000), adriamycin (Scheithauer *et al*, 1999, Neri *et al*, 2002), docetaxel (Jacobs, 2002), and oxaliplatin (Louvet *et al*, 2002) led to higher response rates and interesting results in terms of clinical benefit. However, the majority of these studies also reported to have little effect on patient median survival (range 7–8.3 months), and were all complicated by grade III–IV heamatological and gastroenteric toxicity. Only one of these studies (Louvet *et al*, 2002) reported a promising antitumour activity of GEM+oxaliplatin with a response rate of 30.6%, clinical benefit improvement in 40% of cases, and an overall survival of 9.2 months with 36% of the patients being still alive after 1 year. The multidrug combination of GEM with 5-FU was certainly the most investigated for the treatment of this disease. A number of studies have, in fact, tested the combination administering the two drugs by using different modalities (continuous infusion, or bolus, weekly administrations, etc.) at different doses, with or without FA. These studies reported discordant results in terms of clinical responses, time to progression and overall survival and some of them recorded a high degree of therapy related toxicity (Grem, 1996; Hidalgo *et al*, 1997,Hidalgo *et al*, 1999; Michel and Moore, 1997; Berlin *et al*, 1998–99; Schulz *et al*, 1998; Cascinu *et al*, 1999; Ulrich-Pur *et al*, 2000; Cohen *et al*, 2002; Heinemann, 2002; Oettle and Riess, 2002). The results of the only phase III trial comparing the weekly schedules of 5-FU/GEM with GEM alone did not show any advantage of the combination in terms of overall survival, progression-free survival, or response rate (Berlin *et al*, 2002).

The majority of these studies combined the two fluoropyrimidines on empiric clinical bases, and this may have generated the large discordance in the results; in our study, we have instead designed a novel schedule of a combination aimed to maximise the antitumour efficacy of GEM+FUFA, and to reduce the occurrence of side effects taking advantage of the results of previous preclinical studies, pharmacokinetic analyses, and considering the possibility of administering the drugs on an outpatient basis at a low cost. In the first place in fact, our preclinical data suggested that GEM given before 5-FU produces synergistic antitumour (Correale *et al*, 2000) and proapoptotic (Correale *et al*, unpublished results) activity in colon and pancreas carcinoma cell lines *in vitro*, in the second instance, we also considered that GEM pretreatment enhances the systemic exposure of 5-FU in cancer patients by increasing its area under the curve (AUC) and reducing its clearance (Correale *et al*, 2003). We also considered the results of previous phase I–II clinical trials testing the first GEM+FUFA combination in patients with various gastroenteric carcinomas, which suggested a significant antitumour activity in patients with pancreatic carcinoma unfortunately hampered by a high level of gastroenteric toxicity mainly due to the 5-day schedule of 5-FU administration and the weekly administration of GEM (Correale *et al*, 1999,Correale *et al*, 2000). In the novel biweekly schedule, therefore the dose intensity of GEM remained unchanged, and although distributed in two consecutive weeks, the monthly 5-FU dose intensity was increased. With these modifications, our new schedule of treatment definitely improved the patients’ quality of life during the treatment, significantly reduced the toxicity of the combination, and led to a significant rate of objective responses, with a prolonged time to progression and overall survival. These positive results do not seem to be due to a better selection of the patients because, at the time of the enrolment, 11 patients (26.2%) had very advanced local–regional disease, 22 (52.4%) had liver metastases and nine had metastatic sites. It is interesting to note that a number of the patients are still alive 36 months after the diagnosis, that eight patients with a partial response had liver involvement, and two patients subsequently underwent successful partial hepatectomy for liver metastases and are still alive and disease free 18 months later. It is notoriously difficult to compare the median survival data of early phase II trials but, given the clinical results and the toxicity of the studies described above, our findings seem to be very promising. To what extent these patients will benefit in terms of survival from the biweekly treatment with GEM and FUFA in comparison with GEM alone or no treatment at all awaits the results of future prospective randomised trials.
